# Antifibrotic role of low-dose mitomycin-c-induced cellular senescence in trabeculectomy models

**DOI:** 10.1371/journal.pone.0234706

**Published:** 2020-06-23

**Authors:** Le-Tien Lin, Jiann-Torng Chen, Da-Wen Lu, Ming-Cheng Tai, Chang-Min Liang, Ching-Long Chen, Shu-I Pao, Chih-Kang Hsu, Yi-Hao Chen

**Affiliations:** 1 Department of Ophthalmology, Tri-Service General Hospital Songshan Branch, National Defense Medical Center, Taipei, Taiwan, Republic of China; 2 Graduate Institute of Medical Sciences, National Defense Medical Center, Taipei, Taiwan, Republic of China; 3 Department of Ophthalmology, Tri-Service General Hospital, National Defense Medical Center, Taipei, Taiwan, Republic of China; 4 Graduate Institute of Aerospace and Undersea Medicine, National Defense Medical Center, Taipei, Taiwan, Republic of China; University of Hawaii System, UNITED STATES

## Abstract

**Purpose:**

We assessed whether mitomycin-C (MMC) has different antifibrotic mechanisms in trabeculectomy wound healing.

**Methods:**

We identified 2 concentrations of MMC as “low-dose” by using WST-1 assay, Lactic dehydrogenase assay, and fluorescence-activated cell sorting flow cytometry. Senescence-associated β-galactosidase (SA-β-gal) and fibrotic gene expression was examined through immunocytochemistry, flow cytometry, real-time quantitative reverse transcription polymerase chain reaction, Western blotting, zymography, and modified scratch assay in vitro. In vivo, 0.1 mL of MMC or normal saline was injected to Tenon’s capsule before trabeculectomy in a rabbit model. SA-β-gal expression, apoptotic cell death, and collagen deposition in sites treated and not treated with MMC were evaluated using terminal dUTP nick end labeling assay and histochemical staining. Bleb function and intraocular pressure (IOP) levels were examined 3, 7, 14, 21, 28, and 35 days after trabeculectomy.

**Results:**

In vitro, human Tenon’s fibroblast (HTF) senescence was confirmed by observing cell morphologic change, SA-β-gal accumulation, formation of senescence-associated heterochromatin, increased p16^INK4a^ and p21^CIP1/WAF1^ expression, lower percentage of Ki-67-positive cells, and decreased COL1A1 release. Increased expression of α-SMA, *COL1A1*, and Smad2 signaling in TGF-β1-induced stress fibers were passivated in senescent HTFs. In addition, cellular migration enhanced by TGF-β1was inactivated. In vivo, histological examination indicated increased SA-β-gal accumulation, lower apoptosis ratios, and looser collagen deposition in sites treated with 0.2 μM MMC. Low-dose MMC-induced cellular senescence prolonged trabeculectomy bleb survival and reduced IOP levels in a rabbit model.

**Conclusion:**

Low-dose MMC-induced cellular senescence is involved in the antifibrotic mechanism of trabeculectomy wound healing.

## Introduction

Glaucoma is an optic neuropathy that can lead to irreversible blindness [1, 2]. Although increased intraocular pressure (IOP) is not the only cause of visual impairment, current treatments reduce IOP by using topical medications, lasers, or surgical intervention [3] and can arrest glaucoma [4, 5]. Since its introduction by Cairns in 1968, trabeculectomy, which entails creating a direct channel connecting the anterior chamber and the subconjunctival space, is the benchmark among the available glaucoma surgical techniques [6, 7]. However, excessive postoperative scarring at the filtering bleb site can counteract the benefits of trabeculectomy by closing down the artificial route [8–10]. Therefore, minimizing postoperative scarring has become a vital part of trabeculectomy. One of the most effective means of controlling scar formation is the application of mitomycin-C (MMC) [11, 12]. Currently, no satisfactory method has been developed to resolve this problem.

MMC is a chemotherapeutic agent. It acts as an alkylating agent that causes double-stranded DNA cross linking [13]. The effectiveness of MMC in trabeculectomy wound healing is focused on antiproliferative and apoptotic signaling for fibroblasts in Tenon’s capsule and the conjunctiva [14]. Although MMC exerts its antiscarring effect by activating apoptotic signaling pathways in fibroblasts [14], the antifibrosis mechanism in trabeculectomy wound healing is complicated. Some patients who have received filtrating surgery benefit from a low-dose MMC subconjunctival injection [15]. Notably, low-dose MMC induces stress-induced premature senescence (SIPS) in A549 cells [16]. In addition, Jun et. al demonstrated that induced senescent fibroblasts prevented fibrosis in a cutaneous wound-healing model [17]. SIPS is defined as extrinsic senescence, which can be triggered by subcytotoxic stresses such as ultraviolet light, oxidative stress, or cell treatment with DNA-damaging drugs [18, 19]. Intrinsic senescence was first defined in 1961 when Hayflick and Moorhead observed that human fibroblasts ceased proliferation following an extended cultivation [20]. Both types of senescence remain *viable and metabolically active*, *but they are unable to proliferate despite the presence of nutrition and mitogens*. Human Tenon’s fibroblasts (HTFs) play a critical role in the pathogenesis of bleb scarring by differentiating into an activated, profibrotic myofibroblast phenotype [21]. Whether SIPS can be induced by MMC in HTFs and its involvement in the antifibrotic mechanism of trabeculectomy wound healing are noteworthy topics. However, *assessing what role*, *if any*, *SIPS plays in HTFs is difficult in instances when MMC is effective*.

The present study explored conditions in which cellular senescence might be induced by low-dose MMC in vitro and in vivo. Expression of fibrotic genes indicated SIPS in HTFs has antifibrotic effects in a wound-healing model. Furthermore, inhibited collagen production and preserved bleb function resulting from Tenon’s capsule exposure to low-dose MMC was demonstrated in our rabbit trabeculectomy model, thereby improving surgical outcomes. These findings suggest that SIPS induced by low-dose MMC is involved in the antifibrotic mechanism of trabeculectomy wound healing.

## Materials and methods

### Cell culture and treatment

Human Tenon’s capsule tissue samples were obtained anonymously from tissue explants obtained during strabismus or glaucoma filtering surgery at the Tri-Service General Hospital, National Defense Medical Center, Taipei, Taiwan, with the approval of the ethics committee of the National Defense Medical Center and in accordance with the Declaration of Helsinki. The tissues were cut to approximately 2 × 1 mm^2^ under sterile conditions. The samples were then seeded in 60-mm culture dishes and propagated in Dulbecco’s modified Eagle’s medium/nutrient mixture F-12 (DMEM-F12; Gibco, Carlsbad, CA, USA) supplemented with 10% fetal bovine serum (FBS; Gibco, Carlsbad, CA, USA), 100 U/mL penicillin, and 100 μg/mL streptomycin (Sigma-Aldrich, St. Louis, MO, USA) in a 5% CO_2_ humidified atmosphere at 37°C. For series quality control, observations of cell morphology, immunostaining for fibroblast markers, vimentin (Santa Cruz Biotechnology, Santa Cruz, CA, USA) were performed, and growth curves, proliferation, and population doubling times were determined in isolated primary cells. For all experiments, cells from passages 3 to 6 were used. All experiments were performed at least 3 times with similar results. To induce SIPS, HTFs were treated with 0.02 μM or 0.2 μM MMC (Boxter Oncology GmbH, Kantstraße 2, Halle, Germany) for 48 h, after which the cells were cultured for another 3 days. To generate myofibroblasts, HTFs were exposed to 10 ng/mL TGF-β1 (PeproTech, Rocky Hill, NJ, USA) for 24 h.

### Cell viability assay

Cell viability assays were assessed using the ready-to-use Cell Proliferation Reagent 4-[3-(4-iodophenyl)-2-(4-nitrophenyl)-2H-5-tetrazolio]-1,3-benzene disulfonate (WST-1; Roche Diagnostics, Indianapolis, IN, USA) according to the manufacturer’s recommended protocol. Briefly, confluent monolayer HTFs were incubated with various concentrations of MMC in a serum-free medium for 48 h, and then 10 ul of WST-1 reagent was added to the medium of each well. The cells were incubated for 1 h in a humidified atmosphere at 37°C in 5% CO_2_/95% air, shaken thoroughly for 1 min, and the absorbance was read at 450 nm. Background absorbance was measured in wells containing only the dye solution and culture medium. Cell viability data were obtained from at least 3 experiments with at least 6 wells at each concentration in separate 96-well plates. The average optical density value corresponding to the untreated control was defined as 100%. Results are expressed as a percentage of the optical density of the treated cells relative to the untreated control.

### Cytotoxicity assay

Lactic dehydrogenase (LDH) release was measured using the LDH Cytotoxicity Detection kit (Clontech, Mountain View, CA, USA) to evaluate cell membrane integrity in accordance with the manufacturer’s instructions. Briefly, the HTFs were pretreated with various concentrations of MMC in a low-serum (1% FBS) medium for 48 h to minimize the effects of LDH in the serum. After treatment, the cell-free culture supernatant was collected and incubated with the LDH assay solution for 30 min at room temperature. The optical density values were analyzed at 490 nm. The experiment was repeated 3 times, and the results are expressed as the percentage of maximum LDH release, which was calculated as the ratio relative to 100% cell lysis in 1% Triton X-100 (Sigma-Aldrich, St. Louis, MO, USA).

### Apoptosis assay

MMC-induced HTF apoptosis was examined using a flow cytometer. HTFs were incubated with various concentrations of MMC for 24 h. The isolated cells were then stained with fluorescein isothiocyanate-conjugated Annexin V and propidium iodide (PI) by using the Apoptosis Detection Kit (BD Biosciences Pharmingen, San Jose, CA, USA). Briefly, viable cells exclude PI and do not bind Annexin V. Apoptotic and necrotic cells externalize phosphatidylserine on the plasma membrane and are labeled with Annexin V. Apoptotic cells with intact membranes exclude PI, whereas necrotic cells take up PI, which stains nuclear DNA. Therefore, viable cells can be quantified to assess the effect of MMC-induced apoptosis. Duplicates in each sample were run in accordance with the manufacturer instructions. Confluent cultured cells were then washed twice in phosphate buffer saline (PBS) and analyzed through flow cytometry on a fluorescence-activated cell sorting (FACS) flow cytometer (Becton, Dickinson, and Co., Sunnyvale, CA, USA).

### Detection of senescence-associated β-galactosidase activity

Senescence-associated β-galactosidase (SA-β-gal) assay was performed as described previously [22]. Briefly, the SA-β-gal activity was measured using flow cytometric fluorescence detection in vitro. The HTFs were incubated with 5-Dodecanoylaminofluorescein Di-β-D-Galactopyranoside (C12FDG; Thermo Fisher, Eugene, OR, USA), a β-galactosidase substrate that becomes fluorescent after cleavage by the SA-β-gal. The SA-β-gal-positive cells and median fluorescence intensities (MFIs) were detected and quantified through flow cytometry. The MFIs and percentage of SA-β-gal-positive cells were measured separately. β-Galactosidase is a collective name for enzymes that cleave nonreducing β-D-galactose residues from glycoproteins, sphingolipids, and keratan sulfate in β-D-galactosides [23]. SA-β-gal activity can also be cytochemically detected using 5-bromo-4-chloro-3-indolyl-b-D-galactoside (X-gal; Sigma-Aldrich, St. Louis, MO, USA) as a substrate. In cytochemical or histochemical detection of SA-β-gal activity, the HTFs or frozen tissue sections (6 μm thick) were incubated with SA-β-Gal solution (X-gal, 1 mg/mL; citric acid/sodium phosphate, pH 5.8, 40 mM; potassium ferrocyanide, 5 mM; potassium ferricyanide, 5 mM; NaCl, 150 mM; MgCl2, 2 mM) for 12 h at 37°C. After PBS washing, SA-β-gal-positive cells were analyzed under light microscopy.

### Immunocytochemistry

HTFs cells were seeded on Matrigel-coated coverslips (10 mm in diameter) in 6-well tissue culture plates and then incubated in the presence or absence of low-dose MMC for 48 h. For immunocytochemistry, cells were washed thrice with PBS then fixed in 4% paraformaldehyde for 10 min at 37°C. Next, slides were rinsed thrice in PBS and permeabilized with 0.1% Triton X-100 for 10 min at 37°C. Finally, the slides were washed thrice with PBS then blocked for 30 min at 37°C with 5% BSA to prevent nonspecific staining. The rabbit antihuman monoclonal Ki67 antibody (No. A0047, DakoCytomation, Garching, Germany; dilution 1:100) was used as the primary antibody. Alexa Fluor 488 donkey antirabbit immunoglobulin G (IgG) (BioLegend, San Diego, CA, USA; dilution 1:200) was used as secondary antibody. Nuclei were counterstained with 4′,6′-diamidino-2-phenylindole (DAPI; Sigma-Aldrich, St. Louis, MO, USA). Preparations were mounted in 70% glycerol. The protein expressions of Ki67 were examined using a fluorescence microscope (CKX41; Olympus Corporation, Tokyo, Japan).

Senescence-associated heterochromatin foci (SAHF) were first described in 2003 by Narita et al., who observed that the nuclei of senescent cells contain 30 to 50 bright, punctate DNA-stained dense foci that can be easily distinguished from chromatin in normal cells [24]. To visualize SAHF, DAPI was used to stain for DNA. SAHF were documented using fluorescence microscopy [25].

### Measurement of matrix metalloproteinase activity

In the present study, matrix metalloproteinase (MMP) activity in a conditioned medium was measured using zymography. Conditioned medium samples were collected and concentrated using centrifugal filter devices (Millipore, Bedford, MA, USA). Concentrated protein (50–60 μg) was loaded into NOVEX 12% zymogram casein gels (Invitrogen, Carlsbad, CA, USA). Proteins were electrophoresed in Tris/glycine sodium dodecyl sulfate (SDS) running buffer under nondenaturing conditions. Gels were washed thrice in 2.5% Triton X-100 for 30 min at room temperature to remove SDS. Zymograms were subsequently developed during incubation for 48 h at 37°C in zymogram reaction buffer [0.2 mol/L NaCl, 5 mmol/L CaCl_**2**_, 40 mmol/L HCl, 50 mmol/L Tris, 0.02% Brij 35 (pH 7.1)]. Gels were stained with Coomassie Blue. Enzymatic activity was visualized as a clear band against a dark background of stained casein.

### Western blot analysis

The total protein concentration of HTFs cell lysates was measured using the Bicinchoninic Acid Protein assay kit (Pierce, Rockford, IL, USA) with bovine serum albumin as the standard. Equal amounts of the homogenates (20 μg of lysate) were resolved with one-dimensional 10% SDS polyacrylamide gel, separated electrophoretically, and transferred to a polyvinylidene difluoride membrane (Immobilon; Millipore, Rockland, NY, USA). The membranes were blocked with 5% (wt/vol) milk in Tris-buffered saline (50 mM Tris-HCl, pH 7.4, and 150 mM NaCl) containing 0.05% Tween-20 for 1 h at room temperature on a shaking table. The blots were incubated at room temperature for 60 min with a 1:1000 dilution of antibodies against GAPDH (Millipore, Rockland, NY, USA), p21^CIP1/WAF1^ (sc-6246; Santa Cruz Biotechnology, Santa Cruz, CA, USA), p16^INK4a^ (sc-468; Santa Cruz Biotechnology, Santa Cruz, CA, USA), α-SMA (Abcam, Cambridge, UK), and phosphorylated Smad2 (Cell Signaling Technology, Danvers,MA, USA). The membranes were washed and blotted with horseradish peroxidase-conjugated secondary antibody (Jackson ImmunoResearch Laboratories, West Grove, PA, USA; dilution 1:1000) for 1 h at room temperature. The proteins were visualized on X-ray films using the standard enhanced chemiluminescence procedure (enhanced chemiluminescence reagent; Millipore, Billerica, MA, USA), and mean protein levels were determined densitometrically with ImageJ (version 1.46a; provided in the public domain by the National Institutes of Health, Bethesda, MD, USA).

### Closure of scratch wound

A modified scratch wound assay was used to evaluate cell migration as previously described [26, 27]. Briefly, the HTFs pretreated in the presence or absence of low-dose MMC for 48 h were grown to confluence in 6-well plates in complete medium. The confluent monolayers of HTFs were serum starved for 24 h, then a straight scratch was made on the HTFs using a P200 pipette tip (care was taken during scratching process to ensure uniform size and distance for all samples). The HTFs were treated with 10 ng/mL TGF-β1, and the cells’ ability to migrate and close the wound space was assessed through light microscopy at 0 and 24 h after applying the scratch.

### Real-time quantitative reverse transcription polymerase chain reaction analysis

After collection, the HTFs were centrifuged, and the total cellular mRNA was isolated and transcribed into cDNA using TaqMan Reverse Transcription Reagents in accordance with the manufacturer’s instructions. A real-time quantitative reverse transcription polymerase chain reaction (qRT-PCR) was performed using TaqMan Gene Expression Assay probe and primer for Collagen Type I Alpha 1 (*COL1A1*, Hs00164004_m1, Applied Biosystems, CA, USA) and Glyceraldehyde-3-Phosphate Dehydrogenase (*GAPDH*; Hs99999905_m1, Applied Biosystems, CA, USA). Triplicate PCRs were prepared for each sample under the following thermocycling conditions: initiation at 95°C for 10 min, then 40 cycles each consisting of denaturation at 95°C for 15 s and hybridization–elongation at 60°C for 1 minute. The point at which the PCR product was first detected above a fixed threshold (defined the cycle threshold, Ct), was determined for each sample. Relative expression was calculated using the ΔΔCt values, and results were expressed as 2^−ΔΔCt^, where ΔΔCt = (Ct^*COL1A1*^ − Ct^*GAPDH*^)_test_ − (Ct^*COL1A1*^ − Ct^*GAPDH*^)_control_. The *GAPDH* transcript was used as an internal control to calculate the relative expression levels in each sample. The value of each control sample was set at 1 and used to calculate the fold change of *COL1A1* expression in accordance with the manufacturer’s instructions. The qRT-PCR experiments were repeated 3 times [28].

### Animal grouping design

All animal experiments were approved by and conducted under the guidance of the Institutional Animal Care and Use Committee (accredited by the Association for Assessment and Accreditation of Laboratory Animal Care International), National Defense Medical Center, Taipei, Taiwan (No: IACUC-18-247). All animal experiments were performed in compliance with the Association for Research in Vision and Ophthalmology Statement for the Use of Animals in Ophthalmic and Vision Research. New Zealand White rabbits (National Laboratory Animal Center, Nangang, Taipei, Taiwan) weighing between 2 and 3 kg were used in this study. The rabbits were randomly divided into 3 groups: (1) control group, (2) 0.2 μM MMC group, and (3) 200 μM MMC group. We injected approximately 0.1 mL total volume of normal saline, 0.2 μM MMC, or 200 μM MMC into Tenon’s capsule approximately 6 to 7 mm posterior to the limbus and slightly to the side to avoid the superior rectus muscle. The injection of fluid creates a small raised blister at the injection site. We irrigated the conjunctiva with balanced saline solution and then gently spread the injected bolus of MMC around the superior conjunctiva and Tenon’s layer with a muscle hook. The fluid remained contained within the tissue as we spread it. We then made the first incision and proceeded with the trabeculectomy as normal.

### Rabbit trabeculectomy model and euthanasia

We conducted a modified trabeculectomy that used a cannula to maintain a patent scleral tract [29]. The operation was performed on the right eye only. All surgical procedures and examinations were performed under general anesthesia. The rabbits were anesthetized with an intramuscular injection of a combination of 50 mg/kg ketamine (keTAlaR; Pfizer, Hsinchu, Taiwan) and 10 mg/kg xylazine (Rompun; Bayer, Gyeonggi-Do, South Korea). After a rabbit became unconscious, we determined the depth of anesthesia by lightly pinching one foot pad to evaluate the presence of a reflex response. The operation was not performed until weak or no reflex was noted. Corneal analgesia was administered using a drop of topical 0.5% proparacaine hydrochloride ophthalmic solution (Alcaine; Alcon, Puurs, Belgium) before starting the operation. We used an eyelid speculum to retract the eyelids, and then a limbus-based conjunctival flap was made in the superolateral quadrant of the eye. Blunt dissection was performed using Westcott scissors (AE-5506; ASICO, IL, USA) to undermine the subconjunctival space and Tenon’s capsule. A 20-gauge micro vitreoretinal blade was then used to create a partial-thickness scleral tunnel approximately 2 to 3 mm from the limbus for insertion of a 22-gauge cannula (Becton Dickinson and Company Sparks, MD, USA) into the anterior chamber. The cannula was advanced to the midpupillary area away from the iris. The scleral end of the cannula was then trimmed 1 mm from the scleral tunnel opening and fixed to the sclera with a 10-O nylon suture (Ethicon Inc., Somerville, NJ, USA). The conjunctival incision was closed with a continuous 8-O vicryl (Ethicon Inc., Somerville, NJ, USA) suture. The anterior chamber was reformed with balanced salt solution, and fluid efflux into the subconjunctival space was confirmed.

The euthanasia of rabbits was conducted in CO_2_ chambers. After CO_2_ exposure, rabbits were placed in room air for 20 min to allow for possible recovery [30].

### Evaluation of bleb function

The rabbit eyes were examined in accordance with routine clinical procedures, including examining for the presence of blebs and measuring IOP. Bleb presence was evaluated using an intracameral injection of 0.1 mL of 0.1% trypan blue (Sigma-Aldrich, St. Louis, MO, USA) on postoperative days (PDs) 0, 7, 14, 21, 28, and 35 with a Healon needle [31]. IOP was monitored using an applanation tonometer (TonoVet; Icare Finland, Espoo, Finland) prior to general anesthesia and on PDs 0, 3, 7, 14, 21, 28, and 35. An average of 3 IOP measurements were obtained for each rabbit. Bleb failure was declared after the observer deemed the fluid efflux into the subconjunctival space to have failed in 2 consecutive examinations. The first of the 2 dates was recorded as the endpoint.

### Terminal dUTP nick end labeling assay

Terminal dUTP nick end labeling assay (TUNEL) is a particularly useful technique for studying late-stage apoptosis [32]. To detect MMC-induced subconjunctival apoptosis, TUNEL assay was performed by using the TUNEL Apoptosis Detection kit (Millipore, Bedford, MA, USA) 1 day after trabeculectomy. The subconjunctival cells were stained with DAPI to label nuclei. Images of TUNEL-positive cells were acquired through fluorescence microscopy. The percentage of TUNEL-positive cells was defined as the number of TUNEL-stained cells divided by the number of DAPI-stained cells.

### Histological and immunohistological examinations

Histological examination was performed 21 days after surgery. Eyeballs were enucleated, fixed in 10% neutral buffered formalin solution for 24 h, dissected at the equator, and embedded in paraffin. Anterior segments of eyes containing the cornea, sclera, and conjunctiva were cut sagittally at a thickness of 5 μm, mounted on subbed slides, and dried. The sections were dewaxed in xylene, rehydrated in alcohol, and stained with haematoxylin, eosin, and Masson’s trichrome stain. For SA-β-gal immunohistochemical staining, the enucleated eyeballs were embedded in an optimal cutting temperature compound (Thermo Fisher, Runcorn, UK), and the tissue block was immediately placed in a −80°C freezer. After 12 h of freezing, the temperature of the tissue block was allowed to equilibrate to the temperature of the cryostat (−20°C). The tissue block was placed on the cryostat specimen disk. The positioning of the block was adjusted to align the block with the knife blade. Subsequently, serial axial cryostat sections (6 μm thick) were cut using the microtome portion of the cryostat. Sections were placed on a Fisher Superfrost slide, and slides were fixed by immersion in cold acetone (−20°C) for 2 min, and air dried at room temperature. SA-β-Gal solution staining then proceeded overnight at room temperature [22, 33].

### Statistical methods

Descriptive statistics are expressed as mean ± standard error of the mean of at least 3 independent experiments. Normally distributed continuous variables were compared using a one-way analysis of variance. All statistical assessments were two-sided and evaluated at a significance level of 0.05. Statistical analyses were performed using GraphPad Prism 6.0 (GraphPad Software, Inc., San Diego, CA, USA).

## Results

### Effect of MMC on HTF viability and toxicity

**Fig 1:**
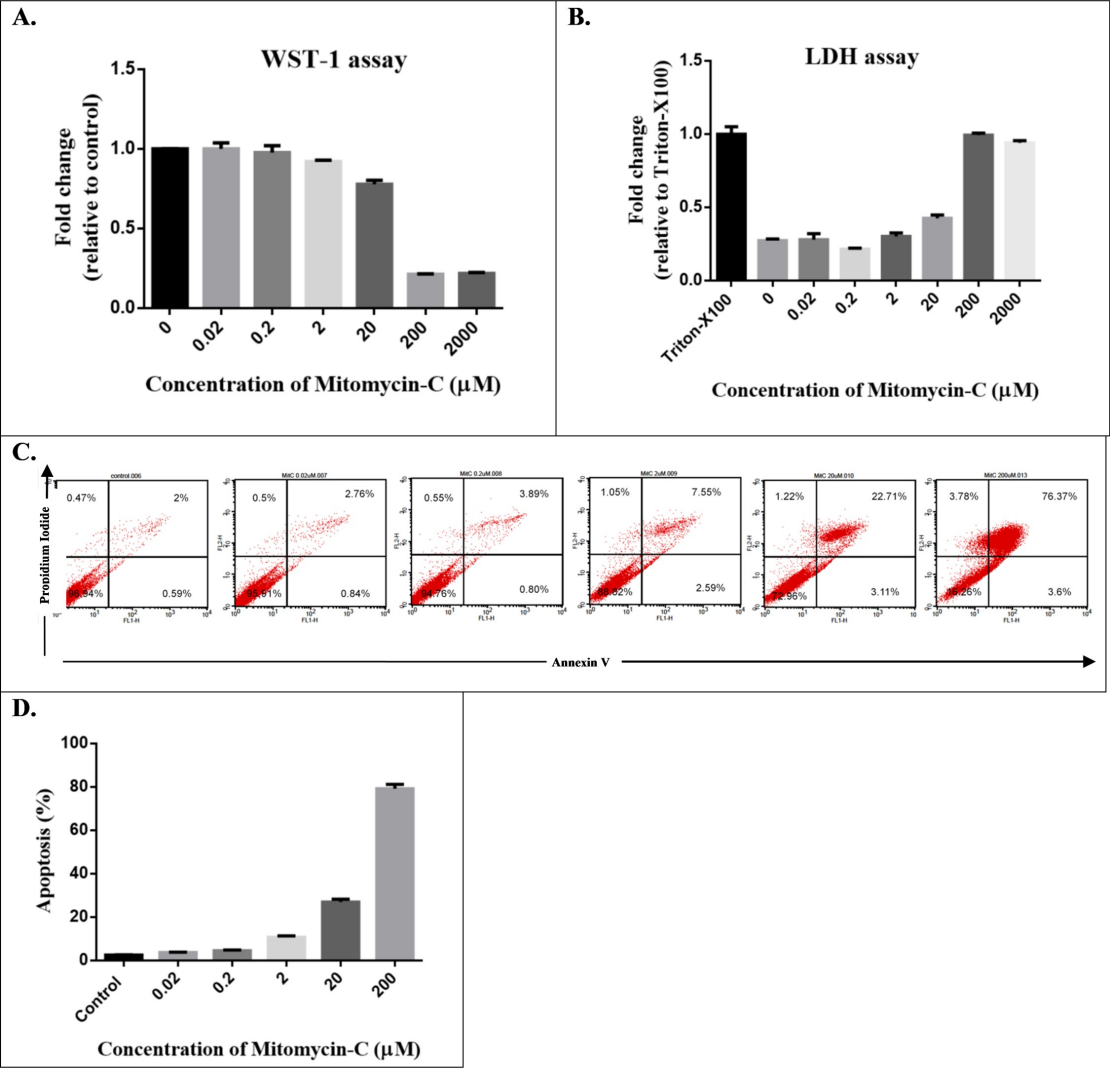
(A) Cell viability was determined using the WST-1 assay. (B) Cytotoxicity of mitomycin-C (MMC) was observed using lactic dehydrogenase assays. (C) MMC-induced apoptotic cell death was determined through fluorescence-activated cell sorting flow cytometry. (D) Bar charts presenting quantitative data of the average of 3 independent flow cytometry experiments in HTFs. When exposing HTFs to various concentrations of MMC, the number of dead cells increased in a dose-dependent manner.

The WST-1 assay was used to determine the viability of HTFs in various concentrations of MMC, whereas the LDH assay was used to measure MMC-induced HTF cytotoxicity (Fig 1A and 1B). The half inhibitory ability of MMC on HTF proliferation was observed at concentrations of 20 to 200 μM. Half cytotoxicity of MMC on HTFs was observed at concentrations of 20 to 200 μM. Furthermore, we used FACS flow cytometry to assess MMC-induced apoptotic cell death. Evidence of apoptosis and secondary necrosis in HTFs was observed 24 h after MMC exposure. Exposing HTFs to various concentrations of MMC led to dose-dependent increases in the number of dead cells (Fig 1C and 1D). The apoptosis rates (% apoptosis and % secondary necrosis) of HTFs significantly increased when exposed to more than 2 μM MMC (Fig 1C and 1D). Therefore, concentrations of 0.02 and 0.2 μM were selected as low-dose MMC in the present study.

### Cellular senescence induced by low-dose MMC in vitro

**Fig 2:**
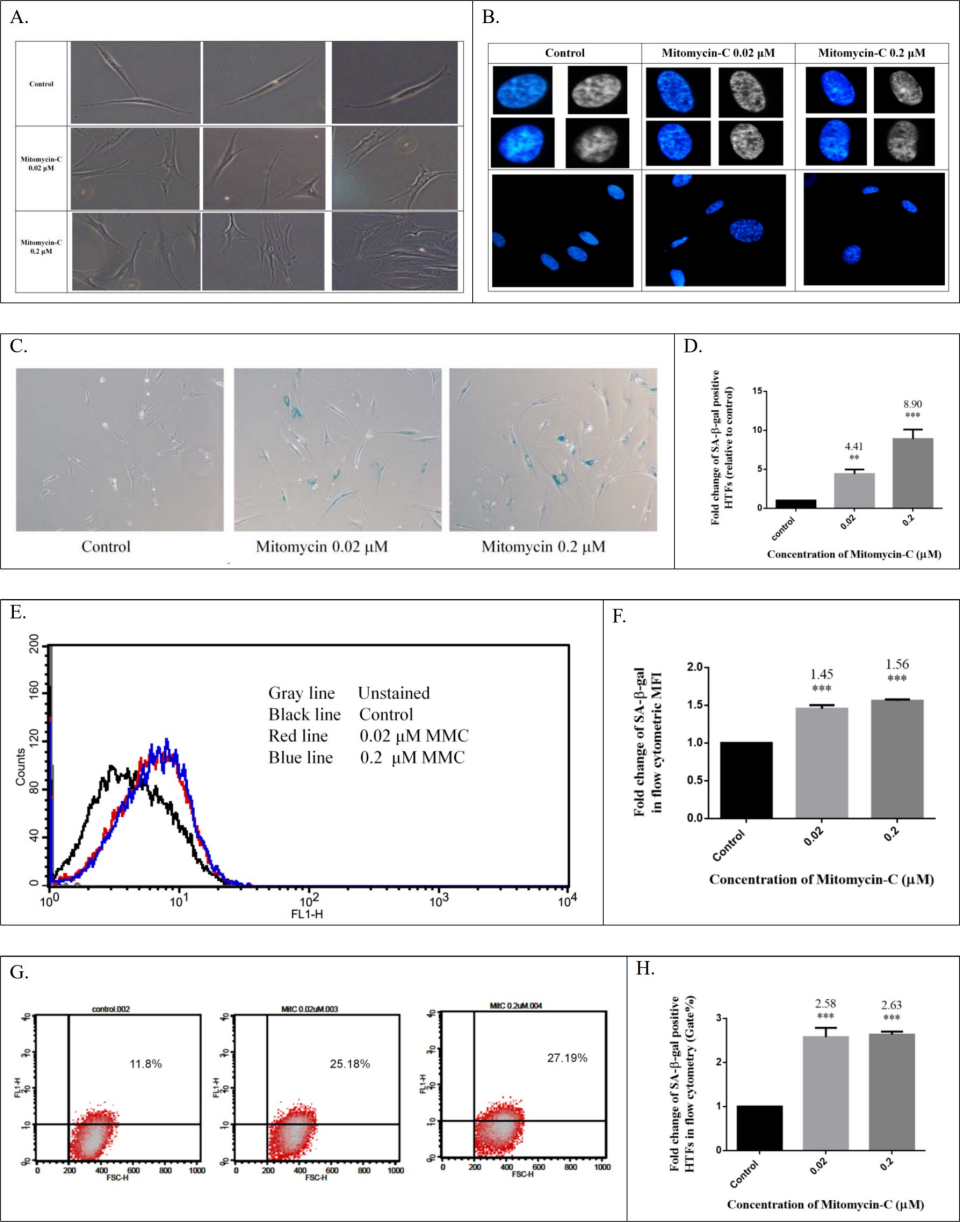
(A) Low-dose mitomycin-C (MMC)-induced senescent HTFs lost their original fibroblastic shape by acquiring an enlarged, flattened morphology with increased nucleus and cell size compared with the control group. (B) Formation of SAHF was noted in HTFs treated with low-dose MMC. SAHF, as identified by immuno- fluorescence microscopy upon chromatin staining with 4′,6′-diamidino-2-phenylindole. (C, D) Cytochemical staining of SA-β-gal activity in the HTFs treated with low-dose MMC was significantly higher than that of the control group. (E, F) Flow cytometric median fluorescence intensity detection revealed that the relative activity was significantly higher in HTFs treated with low-dose MMC. (G, H) Flow cytometric fluorescence detection revealed that the percentage of SA-β-gal positive cells was higher in low-dose MMC-induced senescent HTFs. ***P < .001 versus control group.

Morphologic changes are characteristic features of the senescent phenotype that occurs at both the cellular and organism levels. Senescent cells exhibit morphologically flattened and enlarged cell shapes [34]. The HTFs treated with low-dose MMC exhibited enlarged, extended, flattened, and vacuolated morphology accompanied by the loss of elongated, spindle-like properties that were present in the control group, (Fig 2A). Furthermore, cellular senescence is associated with altered chromatin structures [24]. Senescent cells often exhibit strikingly different punctate staining patterns; whereas DNA dyes display overall homogenous staining patterns in cycling or quiescent human cells. These nuclear DNA domains densely stained with DAPI are known as SAHF [24]. *As illustrated in Fig 2B,* DAPI staining revealed punctate staining (SAHF formation) in the HTFs treated with low-dose MMC, whereas control HTFs exhibited diffuse staining across the cell nuclei. In addition, overexpression of SA-β-gal has been widely used as a marker of cellular senescence [35]. Here, we measure SA-β-gal activity by using cytochemical staining and flow cytometric fluorescence detection [22]. As illustrated in Fig 2C and 2D, cytochemical staining of SA-β-gal in the HTFs treated with low-dose MMC was significantly higher than that in the control group. In the measurement of SA-β-gal activity by using fluorescence detection, the SA-β-gal-positive cells and MFIs were detected and quantified using flow cytometry. In the 0.02 μM and 0.2 μM groups, the MFIs of SA-β-gal were 1.45 and 1.56 fold greater, respectively, than those in the control group (Fig 2E and 2F). In addition, percentages of SA-β-gal-positive cells were 2.58 and 2.63 fold greater, respectively, than those of the control group (Fig 2G and 2H). These findings indicate that low-dose MMC could induce HTF senescence.

### Biomarkers of HTFs treated with low-dose MMC

**Fig 3:**
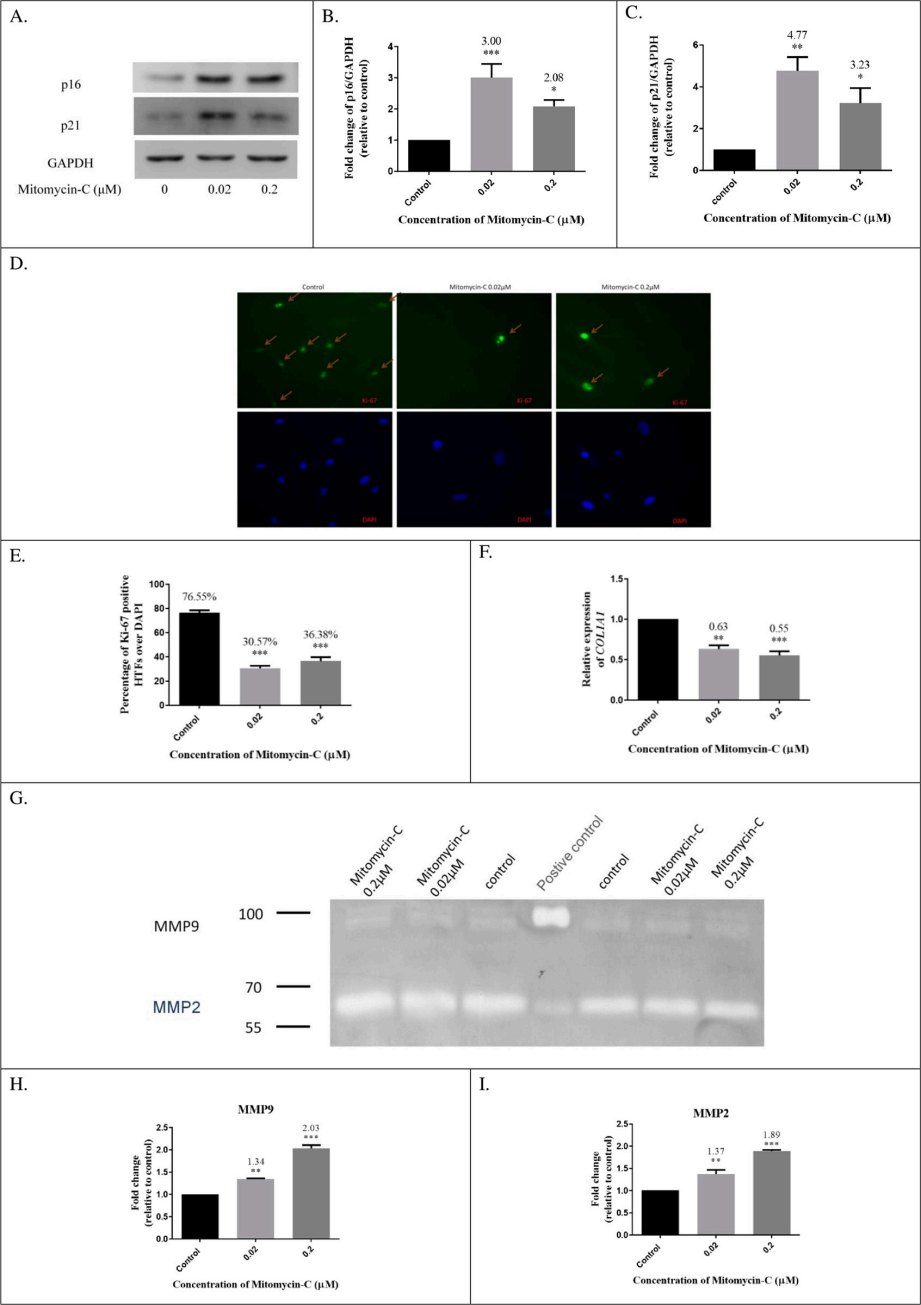
Biomarker performance in low-dose mitomycin-C (MMC)-induced senescent HTFs. (A-C) Western blotting indicated increased p16^INK4a^ and p21^CIP1/WAF1^ expression in senescent HTFs. Western blotting was performed with proteins extracted 3 days after exposure to low-dose MMC for 48 h, using GAPDH protein level as a reference. (D, E) The number of Ki-67-positive cells was counted as a percentage of 4′,6′-diamidino-2-phenylindole-positive nuclei in 5 randomly selected high-power fields. A majority of HTFs treated with low-dose MMC were negative for Ki-67 in immunolabeling. (F) The transcript levels of the *COL1A1* gene decreased in senescent HTFs and measured using qRT-PCR. (G-I) Gelatin zymography indicated that senescent HTFs upregulated expression of MMP-2 and MMP-9 3 days after exposure to low-dose MMC for 48 h. ***P < .001, **P < .01, *P < .05 versus control group.

In addition to morphologic changes, senescent cells exhibit cell cycle arrest. The well-known cyclin-dependent kinase (CDK) inhibitors p16^INK4a^ and p21^CIP1/WAF1^ are the main drivers of cell cycle arrest observed in cellular senescence [36]. We detected p16^INK4a^ and p21^CIP1/WAF1^ expression by using Western blotting. The expression of p16^INK4a^ increased 3.00 and 2.08 fold in the 0.02 μM and 0.2 μM groups, respectively when compared with the control cells (Fig 3A and 3B). Furthermore, p21^CIP1/WAF1^ expression increased by 4.77 and 2.33 fold in the 0.02 μM and 0.2 μM group, respectively compared with the control group (Fig 3A and 3C). To evaluate the HTF proliferation rate, we further performed immunolabeling with anti-Ki67 antibodies in cells from 0.02 μM MMC, 0.2 μM MMC, and control groups [37]. In control groups, the majority of nuclei were positive for Ki67, indicating that fibroblasts were proliferating. By contrast, more than 60% of low-dose MMC-induced senescent HTFs were negative for Ki67 (Fig 3D and 3E).

Collagen production by activated HTFs is associated with increased deposition of the extracellular matrix (ECM), leading to tissue fibrosis [38]. Collagen type I, the major component of ECM, is encoded by the *COL1A1* gene. Here, we investigated the transcript levels of the *COL1A1* gene by using qRT-PCR. The *COL1A1* gene expression was decreased to 0.63 and 0.55 fold in the 0.02 μM and 0.2 μM group, respectively compared with the control group (Fig 3F).

The senescence-associated secretory phenotype (SASP) is associated with senescent cells that influence their neighboring cells and microenvironment through cytokines, chemokines, proteases, and growth factors. MMPs are SASP factors and are calcium-dependent zinc-containing endopeptidases that degrade ECM components. Gelatin zymography was performed to analyze MMP2 and MMP9 expression in cells from 0.02 μM MMC, 0.2 μM MMC, and control groups. MMP9 expression increased by 1.34 and 2.03 fold in the 0.02 μM and 0.2 μM group, respectively compared with the control cells (Fig 3G and 3H). MMP2 expression increased by 1.37 and 1.89 fold in the 0.02 μM and 0.2 μM group, respectively compared with the control cells (Fig 3G and 3I).

### Low-dose MMC effects on cellular senescence and apoptosis in vivo

**Fig 4:**
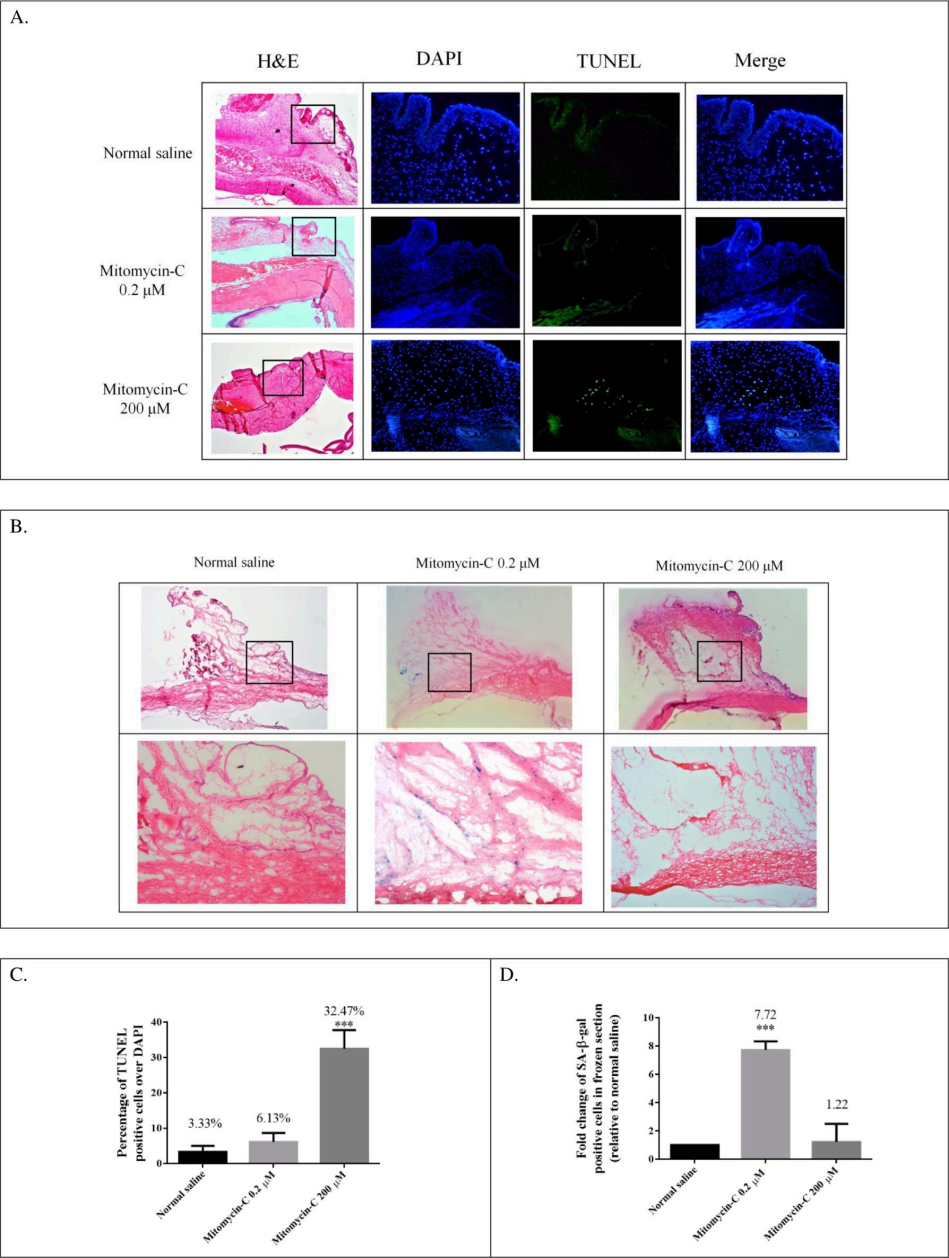
(A,C) Colocalization in TUNEL (apoptosis, green) and 4′,6′-diamidino-2-phenylindole (nuclei, blue) in 3 groups. The percentage of TUNEL-positive cells increased in the 200 μM mitomycin-C (MMC) group and was significantly lower in both control and 0.2 μM MMC groups. (B, D) Frozen section and SA-β-gal staining were used to detect cell senescence in subconjunctival tissues 7 days after trabeculectomy. In the 0.2 μM MMC group, apparent SA-β-gal-positive stained cells were noted. ***P < .001 versus control group.

Because low-dose MMC induces SIPS in HTFs, we further examined whether low-dose MMC could induce subconjunctival cellular senescence in a rabbit trabeculectomy model. To demonstrate that relatively high-dose MMC results in subconjunctival cellular apoptosis and low-dose MMC induces cellular senescence in vivo, we used 200 μM MMC as the “high dose” and 0.2 μM as the “low dose.” The rabbits were randomly divided into 3 groups: (1) control group, (2) 0.2 μM MMC, and (3) 200 μM MMC. To detect subconjunctival cell apoptosis after Tenon’s capsule fibroblast exposure to MMC, TUNEL analysis was performed. Photomicrographs of TUNEL-positive cells in the fibrous layer of conjunctiva in the 3 groups 24 h after trabeculectomy are presented in Fig 4A. The mean percentages of TUNEL-positive cells were 32.47%, 6.13%, and 3.33% in the 200 μM MMC, 0.2% μM MMC, and normal saline groups, respectively (Fig 4C). Thus, cell apoptosis in the fibrous layer of the conjunctiva could be triggered by 200 μM MMC but not by 0.2 μM MMC. To assess cell senescence induced by low-dose MMC, we further performed the frozen section and SA-β-gal staining on the subconjunctival tissues 7 days after trabeculectomy. As illustrated in Fig 4D, the number of SA-β-gal positive cells was 7.72- and 1.22-fold greater in the 0.2 μM MMC and 200 μM MMC groups, respectively, than that in the normal saline group. Apparent SA-β-gal-positive stained cells were noted in the 0.2 μM MMC group (Fig 4B). In summary, the low-dose MMC could induce cellular senescence but not apoptosis in our designed rabbit trabeculectomy model

### Expression of TGF-β1-induced myofibroblast transdifferentiation in senescent HTFs

**Fig 5:**
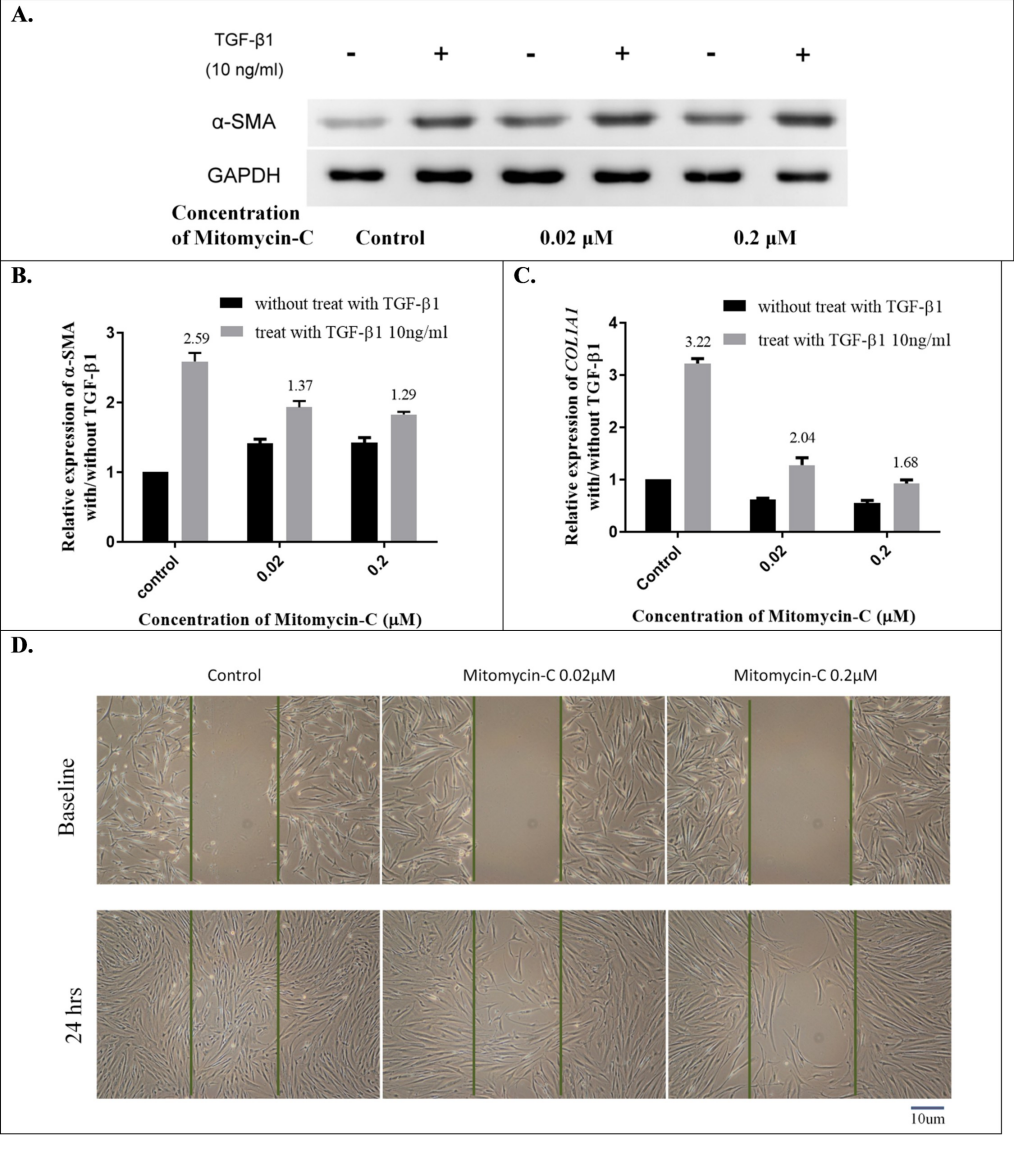
Expression of TGF-β1-stimulated α-SMA, *COL1A1*, and cell migration in senescent HTFs. (A) Expression of TGF-β1-induced α-SMA is passivated on the induced senescent HTFs. (B) The fold changes of TGF-β1-induced α-SMA on the control, 0.02 μM, and 0.2 μM mitomycin-C (MMC) groups were 2.59, 1.37, and 1.29, respectively. (C) The fold changes of TGF-β1-induced *COL1A1* expression in the control, 0.02 μM, and 0.2 μM MMC groups were 3.22, 2.04, and 1.68, respectively. (D) Effects of wound closure in TGF-β1-treated senescent HTFs were quantified with a modified scratch assay. Confluent HTFs were pretreated with or without low-dose MMC, scratched, and then treated with 10 ng/mL TGF-β1. Light microscopy images of HTFs were captured at 0 and 24 h after scratching. The dashed lines outline the central observation area and were added by a masked observer to elucidate the degree of migration. The data are representative of at least 3 independent experiments. Scale bars  =  10 μm.

Activated mesenchymal fibroblasts, called myofibroblasts, are central cellular players in normal wound healing and disappear in the later stages of trabeculectomy [39]. However, they remain in fibrotic diseases and cause excessive scar formation [40–42], including encapsulated blebs in trabeculectomy [39]. The cytokine transforming growth factor beta (TGF-β) drives the conversion of fibroblasts into myofibroblasts, which are abundant in fibrotic lesions and can synthesize ECM [43]. The TGF-β signaling pathway plays a key role in tissue fibrosis, although various fibrogenic signal pathways have been described [44–46]. α-SMA, a myofibroblast marker, increases contractility and stimulates cell migration in TGF-β induced stress fibers [47]. To assess the effect of TGF-β-induced α-SMA expression on the induced senescent HTFs, the 10 ng/mL TGF-β1-induced α-SMA signaling was examined after HTFs incubated with low-dose MMC for 48 h. As illustrated in Fig 5A and 5B, α-SMA expression in HTF cocultured with TGF-β1 was significantly increased (2.59 fold), whereas the fold change of TGF-β1–induced α-SMA signaling on 0.02 μM and 0.2 μM MMC groups was 1.37 and 1.29, respectively. Low-dose MMC-induced HTF senescence had a passivated effect against TGF-β1/α-SMA signaling. One of the TGF-β-related downstream genes is collagen type I. To evaluate the expression of TGF-β1-induced collagen type I production in senescent HTFs, we compared the expression of TGF-β1-induced *COL1A1* from HTFs with or without low-dose MMC. The fold change of TGF-β1-induced *COL1A1* in the control, 0.02 μM and 0.2 μM MMC group were 3.22, 2.04 and 1.68, respectively, suggesting that low-dose MMC-induced HTF senescence could inactivate *COL1A1* expression under TGF-β1 stress (Fig 5C). During the early phase of tissue injury, TGF-β initiates the wound-healing process by recruiting fibroblasts to the site of injury [39, 47]. We assessed the effect of TGF-β-induced cell migration in senescent HTFs by conducting a modified scratch assay. As is evident in Fig 5D, HTFs treated with 0.02 μM or 0.2 μM MMC significantly reduced TGF-β1-mediated cell migration.

### Expression of TGF-β1-induced smad2 signaling in senescent HTFs

**Fig 6:**
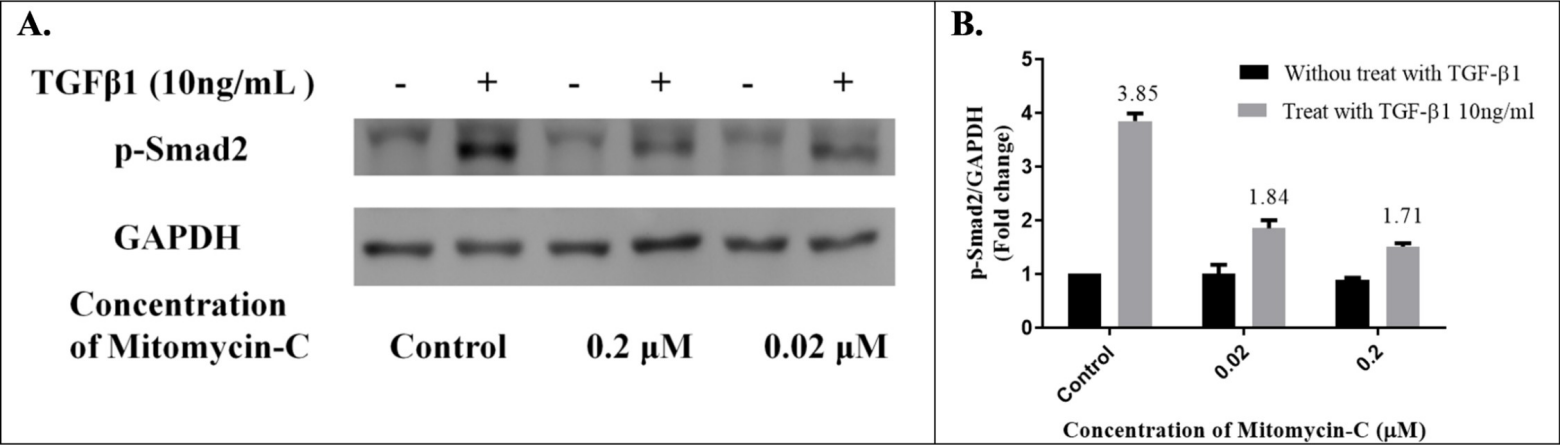
Expression of TGF-β1-induced Smad2 signaling in senescent HTFs. (A) p-Smad2 increased after 10 ng/mL TGF-β1 stimulation in the control group HTFs, but this increase was suppressed in the groups pretreated with 0.02 μM or 0.2 μM mitomycin-C (MMC). (B) The fold change of TGF-β1-induced Smad2 signaling in the control, 0.02 μM and 0.2 μM MMC groups were 3.85, 1.84, and 1.71, respectively.

Phosphorylation of Smad and its subsequent nuclear translocation are critical steps in TGF-β1 signaling [48]. To evaluate the effect of TGF-β1-induced phosphorylation of Smad2 on low-dose MMC-induced senescent HTFs, we pretreated HTFs with low-dose MMC for 48 h. Then, the ability of 10 ng/mL TGF-β1 to induce Smad2 signaling was examined. As illustrated in Fig 6, the Smad2 signal was strongly activated by TGF-β1 in the control group (3.85 fold change), and the signal was relatively inactivated in the cells treated with low-dose MMC. The fold change of TGF-β1-induced Smad2 signaling on the 0.02 μM and 0.2 μM MMC groups was 1.84 and 1.71, respectively; indicating that low-dose MMC-induced HTF senescence had a passivated effect against TGF-β1–Smad signaling.

### Preoperative subconjunctival low-dose MMC injection in a rabbit trabeculectomy model

**Fig 7:**
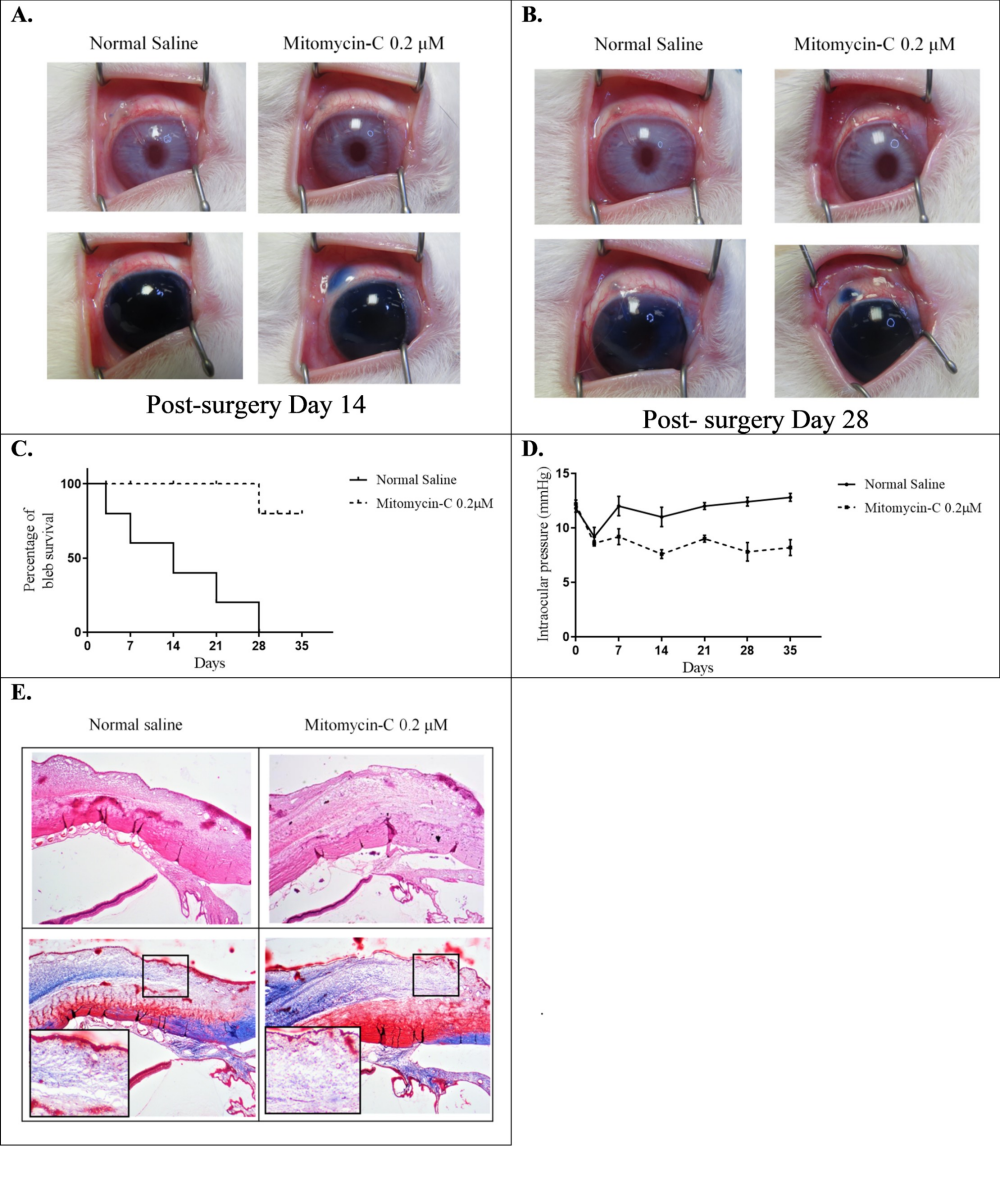
Effects of preoperative subconjunctival low-dose MMC injection in IOP maintenance and bleb survival in the rabbit trabeculectomy model. (A, B) Bleb appearance in both groups on postoperative days (PDs) 14 and 28. (C) Kaplan–Meier bleb survival plot of bleb survival duration in both groups. (D) IOP changes in the two groups over the experimental period. (E) Collagen deposition observed through Masson’s trichrome staining on PD 21 is indicated in blue (area within black lines).

As illustrated in Fig 4, subconjunctival cellular senescence, but not apoptosis, could be induced through low-dose MMC injections in our rabbit trabeculectomy model. The effects of subconjunctival cellular senescence should be further clarified in vivo. Bleb function was evaluated by using an intracameral injection of 0.1 mL of 0.1% trypan blue on PDs 0, 7, 14, 21, and 28 with a Healon needle [31]. As illustrated in Fig 7A and 7B, bleb function after trabeculectomy was significantly improved in the 0.2 μM MMC group compared with the normal saline group. In addition, the Kaplan–Meier survival curve indicates prolonged bleb survival in the 0.2 μM MMC group compared with the normal saline group (Fig 7C). Glaucoma surgery is designed to reduce the IOP. To evaluate the effect of subconjunctival cellular senescence in the animal trabeculectomy model, we measured the IOP on the designated days. We observed no significant difference in IOP between the two groups at baseline. In both groups, trabeculectomy caused a significant reduction in IOP, which was maintained for 3 days, compared with the IOP observed at baseline. Notably, the IOP in the 0.2 μM MMC group remained at a significantly lower level on PD 35 compared with that in the normal saline group (Fig 7D). Furthermore, Masson’s trichrome straining was used to identify differences in collagen deposition in the subconjunctival tissues in both groups on PD 21. Collagen deposition was much looser and rarer in the 0.2 μM MMC group (Fig 7E). In brief, application of low-dose MMC in the rabbit trabeculectomy model led to loose collagen deposition, bleb survival prolongation, and IOP reduction. These preliminary results provide evidence that low-dose MMC can induce subconjunctival cellular senescence and can exert an antifibrotic effect in vivo in a context-dependent manner.

## Discussion

Glaucoma filtering surgery necessitates conjunctival wound healing to prevent filtering bleb leakage. However, excessive proliferation and aberrant ECM production beneath the conjunctiva remain limiting factors in the surgical success rate [8–10]. Until now, adequately regulating the healing process in the surgical site was difficult. MMC is an antibiotic isolated from gram-negative bacteria *Streptomyces caespitosus* with both antineoplastic and antiproliferative properties. It acts as an alkylating agent that causes double-stranded DNA cross linking and inhibits downstream RNA and protein synthesis. A single intraoperative application has lasting effects on fibroblast proliferation [14]. The antiproliferative effect of MMC may be mediated by the activation of the apoptosis-signaling pathway in conjunctival and Tenon’s capsule fibroblasts [14]. Although MMC-induced apoptosis is predominant, low-dose MMC-induced irreversible senescence in A549 cells [16] is of interest to us. Cellular senescence is a recognized mechanism of tumor suppression [49]. However, Jun et al. demonstrated that induced senescent fibroblasts increase antifibrotic gene expression to dampen fibrosis in cutaneous wound healing [17]. In vitro, we treated HTFs with different concentrations of MMC and used WST-1 assay, LDH assay, and FACS flow cytometric analysis to select 2 MMC concentrations as “low doses” that would not induce significant apoptosis in HTFs. As expected, HTFs treated with low-dose MMC exhibited enlarged, extended, flattened, and vacuolated cell morphology, accumulated SA-β-gal, and formed SAHF. In addition to the aforementioned typical distinctive phenotypic changes in cellular senescence, the HTFs treated with low-dose MMC also exhibited a lower percentage of Ki-67-positive proliferative cells, enhanced MMP2 and MMP9 expression, reduced *COL1A1* expression, and upregulated numerous secreted proteins such as p16^INK4a^ and p21^CIP1/WAF1^, which are main components in senescence signaling [36].

The nuclear expression of the Ki67 protein can be used as a cellular marker for cell proliferation [37]. Ki67 is present during all active phases of the cell cycle (G1, S, G2, and M phases) but is absent in resting cells (G0 phase). Collagen type I, the major protein component of the ECM, is synthesized as a procollagen whose triple helix comprises one alpha2 chain and two alpha1 chains, which are encoded by *COL1A2* and *COL1A1*, respectively [50]. The two polypeptide chains are uniformly synthesized in coordination at a rate of 1:2 [50]. The role of the collagen type I gene in normal wound healing as well as in fibrosis has been extensively researched [51, 52]. The CDK inhibitors p16^INK4a^ and p21^CIP1/WAF1^ are efficient inducers of cell cycle arrest [36]. A study demonstrated that adenovirus-mediated p21^CIP1/WAF1^ can prevent fibroproliferation in a rabbit trabeculectomy model [53]. Our findings indicate that senescent HTFs exhibit lower Ki-67-positive percentages, reduce *CoL1A1* expression, and increase p16^INK4a^ and p21^CIP1/WAF1^ release.

Scar formation is a normal wound healing process that provides temporary strength to damaged tissue [54]. With time, the *normal scar* is gradually remodeled during the *healing process*. Defects in the resolution of a normal scar can lead to excessive accumulation of ECM in the tissue, causing pathological scarring. MMPs belong to a larger family of cell-migration-associated proteases that degrade components of the ECM. Several studies have focused on MMP inhibition as a potential antiscarring strategy in glaucoma filtration surgery [55–57]. By contrast, MMP9 inhibits cell proliferation and delays corneal wound healing [58]. These findings suggest that MMPs can either reduce or promote fibrosis [59]. This is a complex topic in which differences in wound modeling may unexpectedly change the result, leading to potentially conflicting conclusions. A study demonstrated that *MMP* genes were overexpressed in senescent human fibroblasts [60]. Consistent with this, the present study demonstrated that MMP2 and MMP9 expression increased in senescent HTFs (Fig 3G–3I).

Several signaling pathways are involved in fibrosis responses, wherein TGF-β–Smad signaling is considered to play a key role in wound healing after trabeculectomy [61, 62]. TGF-β expression increases in response to injury. Numerous studies have indicated that the TGF-β pathway regulates cells migration; induces myofibroblasts to synthesize ECM, and progresses tissue fibrogenesis [63, 64]. In addition, TGF-β overexpression is associated with scarred filtering blebs that contribute to failure of glaucoma filtering surgery in rats [65]. Therefore, to evaluate the effect of TGF-β-induced myofibroblast transdifferentiation, we measured cell migration and expression of α-SMA and *COL1A1* in senescent HTFs. All of the results indicated that senescent HTFs significantly passivated the TGF-β1-induced functional changes. Furthermore, TGF-β1-induced Smad2 phosphorylation decreased. Myofibroblasts play a central role in tissue remodeling after injury and are key drivers of excess scarring [66]. As illustrated in Fig 5A and 5B, senescent HTFs had a passivated effect against TGF-β1–α-SMA signaling, but α-SMA expression was more significant in senescent HTFs, corresponding with the observation by Hecker et al [67]. Notably, senescent cells are believed to downregulate ECM-component-encoding genes and upregulate ECM-degrading enzymes, such as MMP. Myofibroblasts are the primary ECM-secreting cells during wound healing [68]. The effects of the two types of cells on the ECM have been inconsistently reported [17, 68]. Moreover, aging-related senescence seems to be different from SIPS, and this further contributes to the inconsistency [69]. For example, Jun et al. demonstrated that SIPS-fibroblasts prevented fibrosis in a cutaneous wound healing model [17]. However, Huang et al. revealed that aging-related senescent fibroblasts increased fibrotic responses in old mice [70]. In addition, Mellone et al. indicated that senescent fibroblasts represented a special subgroup of SMA-positive myofibroblasts with a contractile phenotype but without the capability to produce ECM [71]. Although further research is necessary, our findings support the notion that senescent HTFs weaken the fibroproliferation response in vitro.

We designed a rabbit trabeculectomy model to examine whether low-dose MMC can induce subconjunctival cellular senescence and assess the outcome of filtering surgery by exposing Tenon’s capsule to 0.2 μM MMC. Notably, the concentration used as low-dose MMC here is much lower than that of a previous study [15], which demonstrated that 150 μM MMC can inhibit subconjunctival fibroblast proliferation. Histological examination indicated that low-dose MMC could induce subconjunctival cellular senescence but not apoptosis in our rabbit model. In Masson’s trichrome staining, collagen expression in the site treated with low-dose MMC had much looser and rarer deposition. In addition, low-dose MMC-induced cell senescence prolonged the survival of trabeculectomy blebs and significantly reduced IOP levels. These preliminary results suggest that application of low-dose MMC can improve the prognosis of trabeculectomy through the antifibrotic effect of cellular senescence. However, further evidence is necessary before preoperative low-dose MMC subconjunctival injection can be used in clinical settings [72]. First, the efficacy and safety of a recommended MMC dose range for humans should be further investigated. Second, in addition to the TGF-β–Smad signaling pathway, several signaling pathways such as MAPK and PI3K-Akt are involved in fibrogenesis-associated TGF-β signaling [73,74]. Exploring which other signaling pathways are involved in scarred filtering bleb formation and the antifibrotic effect of SIPS on these pathways is apealing.

**Fig 8:**
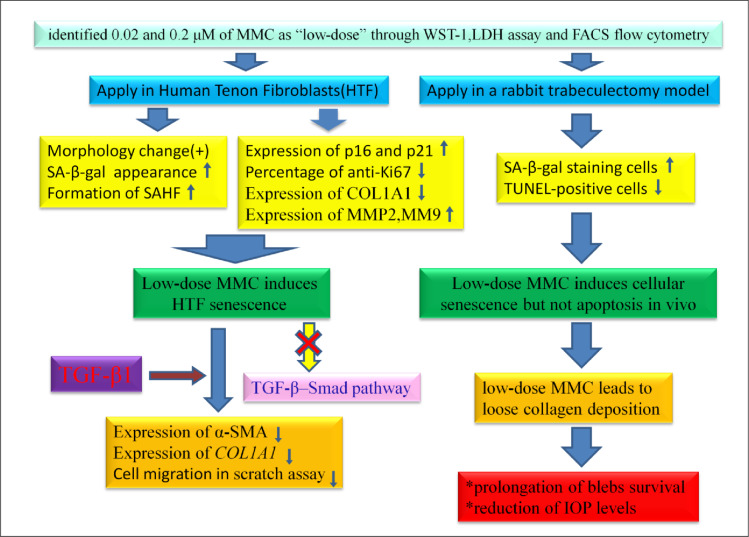
Schematic of application of low-dose MMC to induce cellular senescence (but not apoptosis) and the antifibrotic effect of low-dose MMC in our trabeculectomy models.

In summary, we revealed that low-dose MMC induces SIPS in HTFs, thereby reducing TGF-β1-induced myofibroblast transdifferentiation, cellular migration, and *COL1A1* expression, which are the central events in a fibrotic response. Moreover, a preoperative subconjunctival injection of 0.2 μM MMC reduced postoperative collagen production and preserved bleb function in a rabbit trabeculectomy model, which led to prolonged bleb survival and IOP-lowering effects (Fig 8). We conclude that cellular senescence induced by MMC is involved in the antifibrotic mechanism of trabeculectomy wound healing. A therapeutic strategy may be designed to use MMC at much lower concentrations to induce cellular senescence, thereby minimizing conjunctival scarring and some of the drug-induced toxicity.

## Supporting information

S1 Raw images (PDF)

S1 File (ZIP)
