# Supplementary figures and images for: Antifibrotic role of low-dose mitomycin-c-induced cellular senescence in trabeculectomy models
Source: PLoS One. 2020 Jun 23;15(6):e0234706. doi: 10.1371/journal.pone.0234706 (PMC7310836; doi:10.1371/journal.pone.0234706)

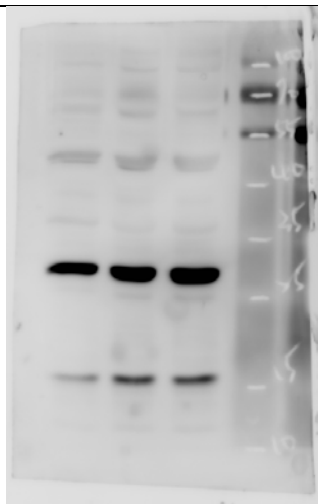

Fig 3A P16 (lower)

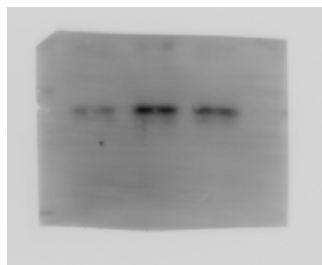

Fig 3A P21

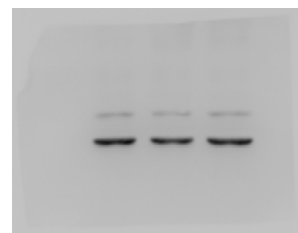

Fig 3A GAPDH

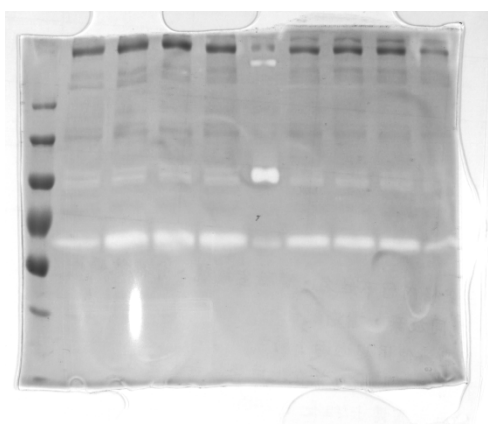

Fig 3G MMP2 + MMP9

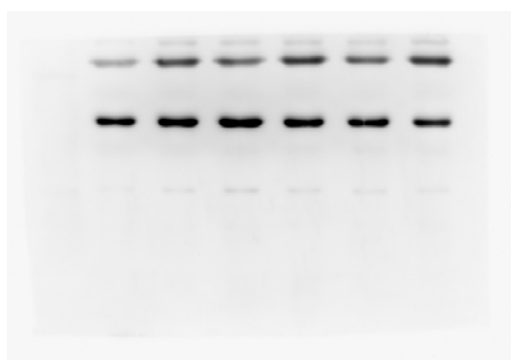

Fig 5A  $\alpha$ -SMA + GAPDH

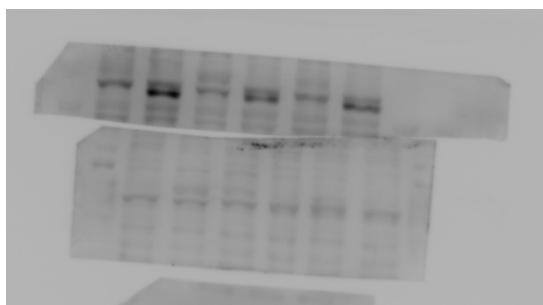

Fig 6A p-Smad2 (upper)

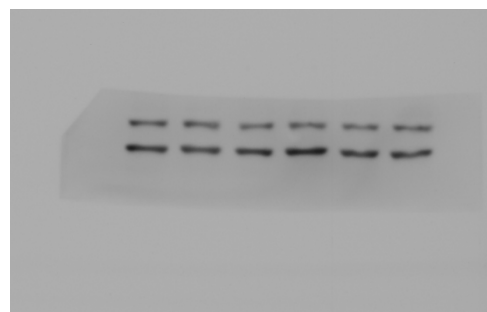

Fig 6A GAPDH

Supplement: S1 Raw images — (PDF) [file pone.0234706.s001.pdf]

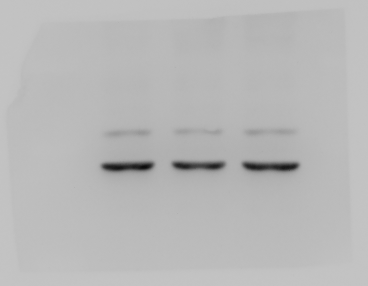

Supplement: S1 File — (ZIP) [file pone.0234706.s002.zip › Supporting Information/P16+P21/GAPDH.tiff]

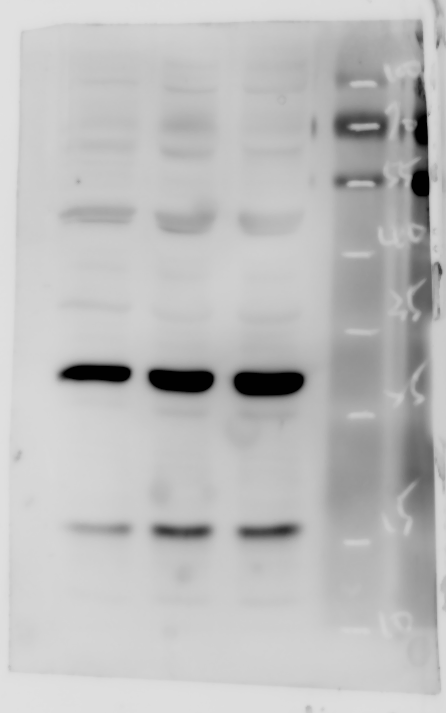

Supplement: S1 File — (ZIP) [file pone.0234706.s002.zip › Supporting Information/P16+P21/p16(lower).tiff]

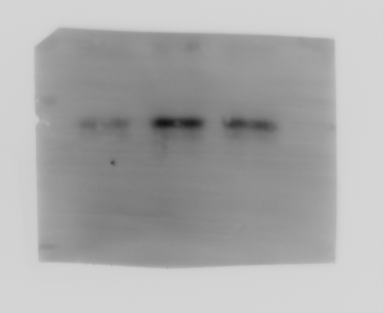

Supplement: S1 File — (ZIP) [file pone.0234706.s002.zip › Supporting Information/P16+P21/p21.tiff]

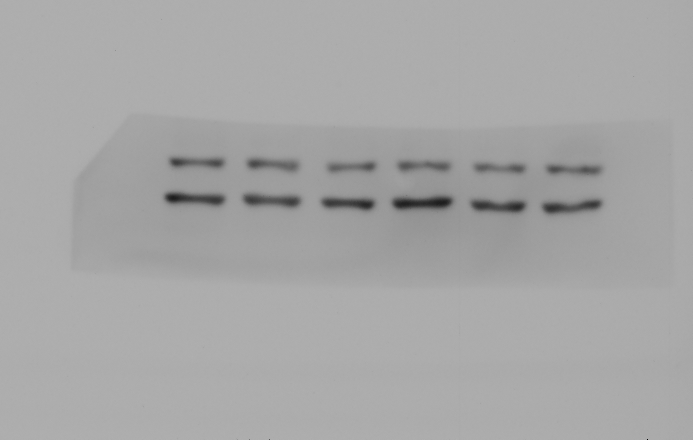

Supplement: S1 File — (ZIP) [file pone.0234706.s002.zip › Supporting Information/p-Smad2/GAPDH.tif]

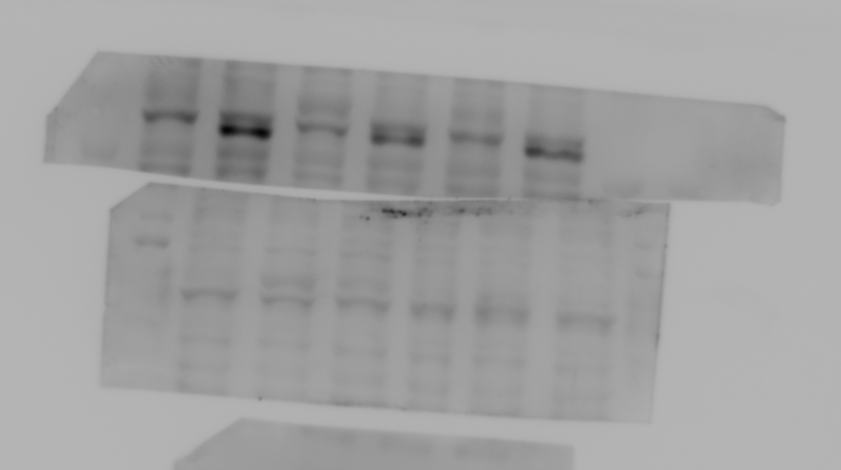

Supplement: S1 File — (ZIP) [file pone.0234706.s002.zip › Supporting Information/p-Smad2/pSmad2(upper).tiff]

| 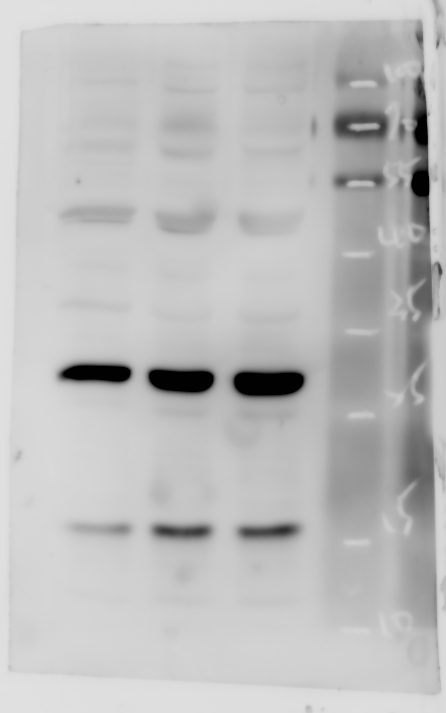  Fig 3A P16 (lower) | 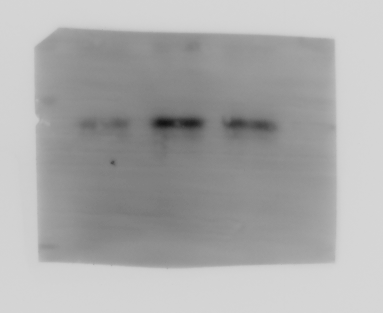  Fig 3A P21 | 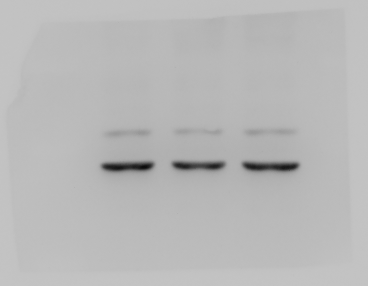  Fig 3A GAPDH |
| --- | --- | --- |

| 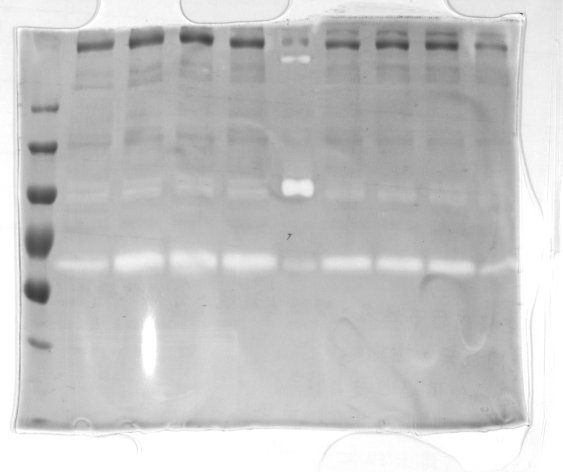  Fig 3G MMP2 + MMP9 |
| --- |

| 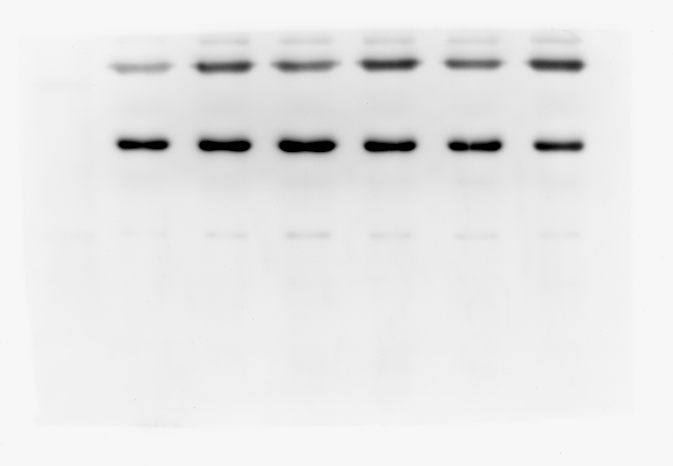  Fig 5A α-SMA + GAPDH |
| --- |

| 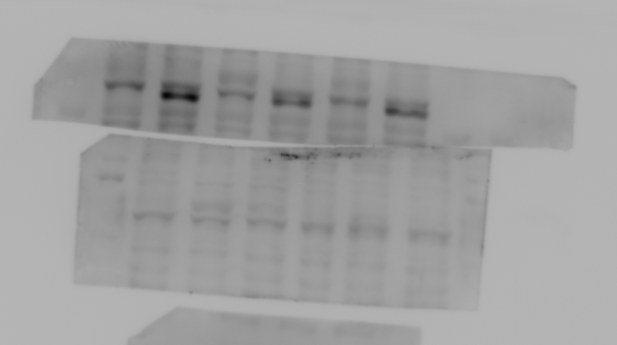  Fig 6A p-Smad2 (uppper) | 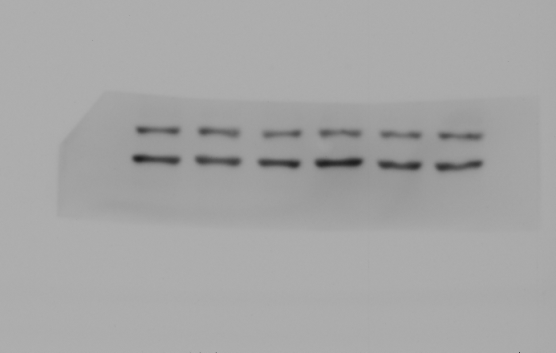  Fig 6A GAPDH |
| --- | --- |

Supplement: S1 File — (ZIP) [file pone.0234706.s002.zip › Supporting Information/S1_raw_images.docx]

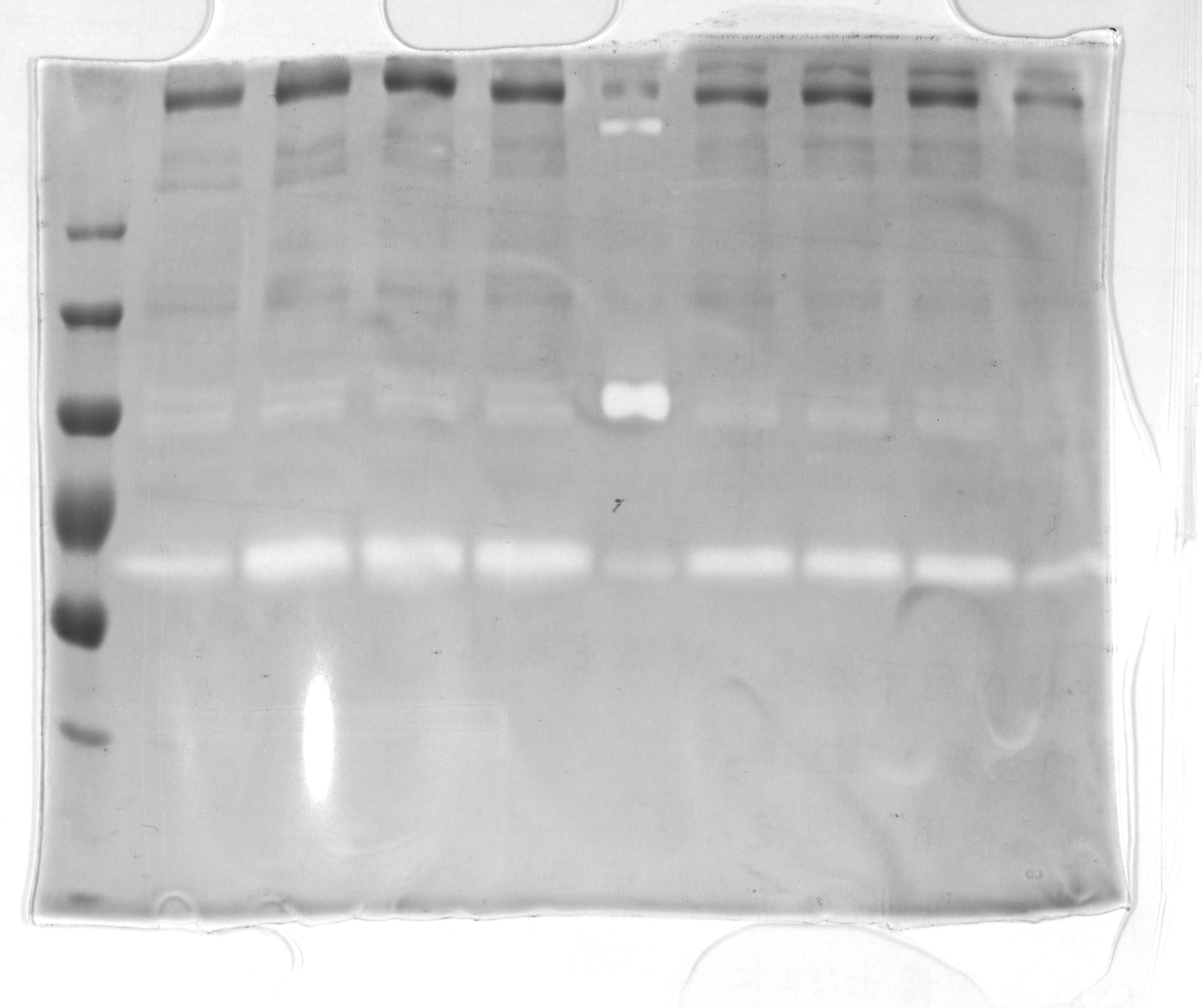

Supplement: S1 File — (ZIP) [file pone.0234706.s002.zip › Supporting Information/Zymography MMP2+MMP9.tiff]

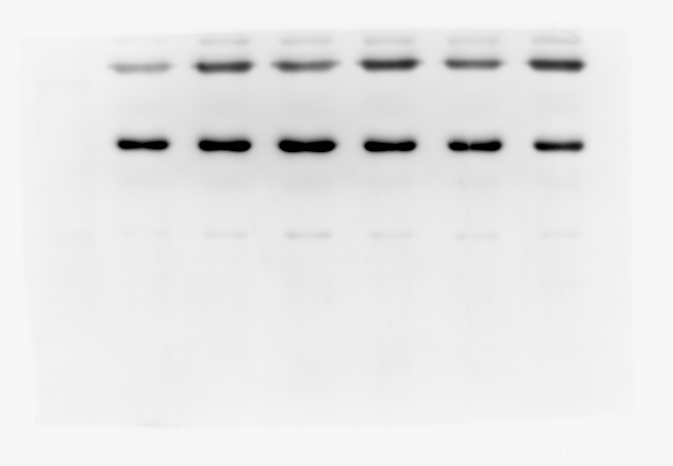

Supplement: S1 File — (ZIP) [file pone.0234706.s002.zip › Supporting Information/ú/-SMA + GAPDH.tiff]
